# Herb-Drug Interactions: Systematic Review, Mechanisms, and Therapies

**DOI:** 10.1155/2015/239150

**Published:** 2015-02-22

**Authors:** Zhong Zuo, Min Huang, Isadore Kanfer, Moses S. S. Chow, William C. S. Cho

**Affiliations:** ^1^School of Pharmacy, Faculty of Medicine, The Chinese University of Hong Kong, Hong Kong; ^2^School of Pharmaceutical Sciences, Sun Yat-sen University, Guang Zhou 510006, China; ^3^Faculty of Pharmacy, Rhodes University, Grahamstown 6140, South Africa; ^4^Center for Advanced Drug Research and Evaluation, College of Pharmacy, Western University of Health Sciences, Pomona, CA 91766, USA; ^5^Department of Clinical Oncology, Queen Elizabeth Hospital, Hong Kong

Since the use of herbs in daily life has become quite prevalent, issues of the safety of coadministration of such products together with Western medicines should be brought into attention. Although the pharmacokinetics and pharmacodynamics of Western medicines are well-known, the activities of any coadministered herbal products have not been well studied due to their complex components and variability. Most reports on drug-drug or herb-drug interactions focus more on pharmacokinetics than on the pharmacodynamics. However, both effects cannot be ignored in practice, especially for interactions that may occur between a single component Western medicine and a multicomponent herbal product. Herb-drug interactions are essential considerations that need to be addressed by undertaking high quality scientific research and conducting thorough systematic literature reviews.

Since our call for submission in January 2014, this special issue on has attracted over forty papers worldwide, ranging from reviews and preclinical research studies to clinical investigations. The ten final accepted articles cover the topics of (1) systematic reviews on the herb-drug interactions of clinically well-known narrow therapeutic index drugs, (2) recent method development and mechanistic studies on herb-drug interactions, and (3) clinical outcomes for commonly seen combination use of herbs and drugs. The five review papers from S. Mogami and T. Hattori, B. Ge et al., Y. K. Fong et al., S. Chen et al., and D. S. Kiefer et al. provided comprehensive updates on the interactions between herbs and Western drugs in the therapeutic areas ranging from oncology, gastrointestinal, and cardiovascular to central nervous system, among which the article entitled “*Interaction of carbamazepine with herbs, dietary supplement, and food: a systematic review*” was featured on http://www.mdlinx.com/ and selected as number 2 on the nursing site at http://www.mdlinx.com/nursing/news-article.cfm/4784813. Other three articles from Q. Chang et al., A. C. Müller et al., and F. Qiu et al., demonstrated the current advanced approaches for the clinical investigations of herb-drug interactions with emphasis on simultaneous monitoring of both pharmacokinetic and pharmacodynamic changes. The two articles by G. Wu and L. Huang et al. illustrated the current innovative mechanistic approaches in studying herb-drug interactions.

We are of the opinion that the current special issue not only highlights the most updated research tools available in the investigation of herb-drug interaction, but also provides some essential skills for the healthcare researchers and practitioners to solve some relevant issues they may encounter in this field.

*Zhong Zuo* *Zhong Zuo* *Min Huang* *Min Huang* *Isadore Kanfer* *Isadore Kanfer* *Moses S. S. Chow* *Moses S. S. Chow* *William C. S. Cho* *William C. S. Cho*

